# Squamous Cell Carcinoma Originating from a Crohn's Enterocutaneous Fistula

**DOI:** 10.1155/2017/1929182

**Published:** 2017-04-09

**Authors:** Bogdan Protyniak, Travis Shutt, Russell Farmer

**Affiliations:** ^1^University of Louisville Division of Colon and Rectal Surgery, Louisville, KY, USA; ^2^Geisinger Health System, Danville, PA, USA; ^3^University of Louisville School of Medicine, Louisville, KY, USA

## Abstract

*Purpose*. Squamous cell carcinoma (SCC) developing within fistulae and chronic wounds in patients with Crohn's disease is a rare phenomenon with few reported studies in the literature. Clinical suspicion for SCC in Crohn's disease patients with chronic painful fistulae is low, leading to delayed diagnosis and treatment. We present a patient with long-standing Crohn's disease complicated by malignant degeneration of an enterocutaneous fistula tract.* Methods*. Workup with MRI, CT, and fistulogram revealed a 7 × 3 cm fistulous connection between a loop of small intestine and the patient's perineum. Biopsies of the fistula tract confirmed the diagnosis of SCC.* Results*. The patient underwent an abdominoperineal resection with positive margins and is currently awaiting radiation therapy.* Conclusions*. This is the second case reported within the English literature of SCC arising from an enterocutaneous fistula in the setting of Crohn's disease. Based on the findings in this case report and others presented in the literature, a high degree of suspicion of malignancy should be present in patients with chronic painful, poor-healing fistulae, especially in the absence of infection.

## 1. Introduction

The development of perineal fistulizing disease in Crohn's is a common occurrence, with a reported incidence of 17–50% [1]. However, malignant degeneration of said fistulae is rare, on the order of less than 1% [2]. A recent systematic review from 1950 to 2008 found 61 published cases of adenocarcinoma and squamous cell carcinoma from perineal fistulae originating in rectum, anus, and vagina [3]. Other publications mention carcinoma arising from a Crohn's colocutaneous fistula [4]. None were reported to have squamous cell carcinoma of a Crohn's fistula tract arising from small intestine. To date, only one other study describes a case of SCC involving a Crohn's enterocutaneous fistula [5]. We present a patient with Crohn's disease who underwent an intersphincteric proctectomy complicated by a chronic enterocutaneous fistula tract that degenerated into SCC.

## 2. Case Presentation

A 37-year-old white female presented to our clinic in 2015 with persistent seropurulent drainage from a perineal wound. She was a chronic smoker with a long history of Crohn's disease and diabetes mellitus. Originally diagnosed with ulcerative colitis at age thirteen, she was managed with numerous courses of steroids, antimetabolites, and 5-aminosalicylates. At 21 years old, she underwent total abdominal colectomy with end ileostomy for toxic megacolon during a twin pregnancy when she stopped her antimetabolite therapy. Her pathology supported a diagnosis of ulcerative colitis and because her rectum was spared, she underwent an ileorectal anastomosis the following year. Over the next three years she developed chronic diarrhea and perianal fistulae. Lower endoscopy revealed friable, heaped-up mucosa, and areas of fibrosis consistent with Crohn's disease. This necessitated a completion proctectomy and ileal pouch-anal anastomosis with a diverting loop ileostomy. Rectal pathology was consistent with active Crohn's disease. She had an uneventful recovery and closure of her ileostomy after a negative contrast enema six months following her surgery.

At 27 years old, the patient started to develop multiple perianal fistulae and abscesses. Treatment consisted of surgical drainage using setons and infliximab. Despite aggressive medical management, her symptoms continued to progress and she required excision of her ileal pouch-anal anastomosis, intersphincteric proctectomy, and end ileostomy the same year. No dysplasia was noted within the specimen, including the endoanal mucosa. Her perineal wound never healed completely, resulting in a chronic draining sinus. She was referred to plastic surgery for tissue flap reconstruction multiple times but was uncooperative and refused to quit smoking.

**Figure 1:**
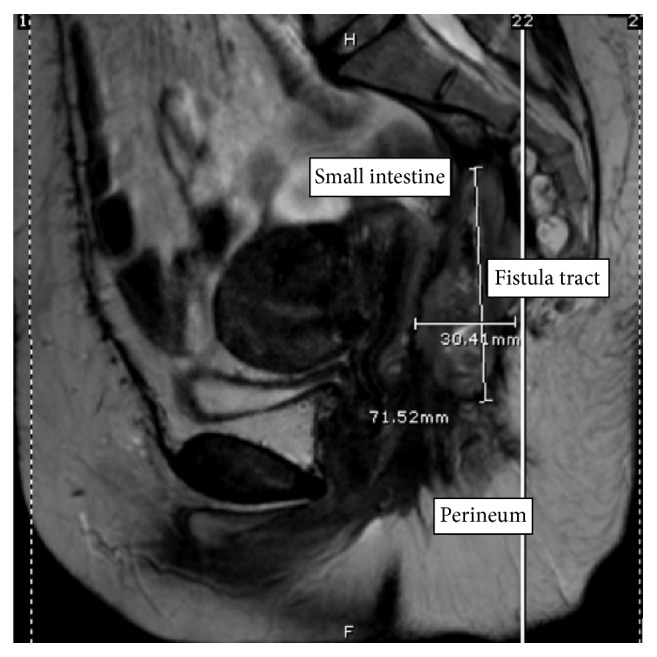
Sagittal view MRI of pelvis demonstrating 7 × 3 cm tract from perineum to presacral space.

**Figure 2:**
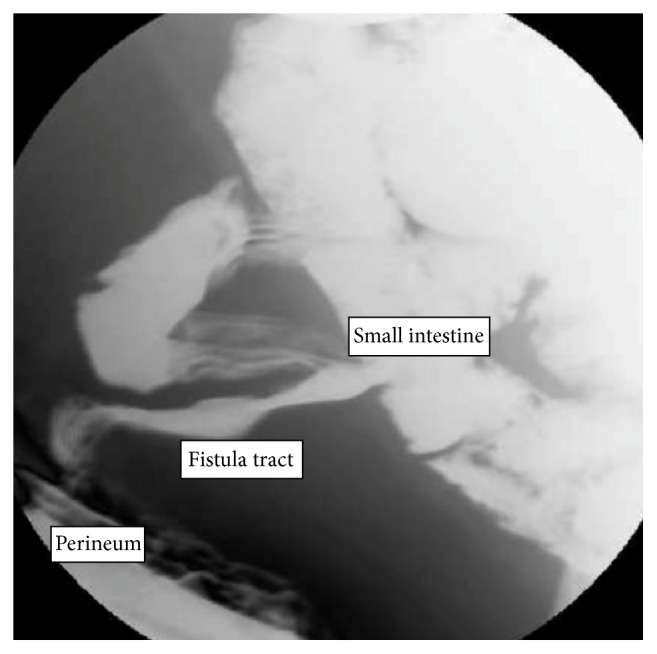
Fistulogram showing passage of contrast through the fistula on the left portion of the image to the small bowel on the right side.

After being lost to follow-up, the patient presented in 2015 with a perineal wound draining seropurulent, sometimes green fluid. An exam under anesthesia revealed a tract in the mid-portion of the perineum extending deep posterior towards the sacrum approximately eight cm. There was no initial evidence of abscess or communication with the uterus, vagina, or bowel. Biopsies showed invasive squamous cell carcinoma. A PET-CT of her chest, abdomen, and pelvis discovered a thick-walled tract 10 cm in length extending from the anus to the presacral space with increased FDG uptake of 12.7. There was an adjacent bowel loop at the tip of the tract without a discernable fat plane. An MRI was obtained, showing a 7 × 3 cm soft tissue mass with a central cavity in the presacral space without associated lymphadenopathy or vaginal/vesical invasion (Figure 1). Finally, a fistulogram was performed and was conclusive for an enterocutaneous fistula (Figure 2).

She underwent an exploratory laparotomy, small bowel resection, takedown of enterocutaneous fistula, and resection of the SCC of the pelvis. The neoplasm was resected to grossly no visible disease and intraoperative frozen margins were negative. The fistula extended from mid-jejunum to the perineum with extensive pelvic inflammatory change and near-complete occlusion of the pelvic outlet. There was complete immobility of the pelvic tissues, resulting in a right ureteral injury, intraoperative hypotension, and estimated 2 L blood loss. These complications resulted in a temporary abdominal closure followed by a nephrostomy tube. During the subsequent operation on postoperative day 2, she had ureteral reconstruction using a Boari bladder flap, a gracilis flap to cover the perineum, and definitive abdominal closure. Final pathology revealed the pelvic mass to be invasive squamous cell carcinoma with positive posterior deep margin. Reexcision was not attempted given that all visible disease was resected at initial operation. She has since recovered from her surgery and will be treated with pelvic radiotherapy for microscopically positive margin.

## 3. Discussion

Perineal fistulizing disease is a common, chronic, and often treatment-refractory complication of Crohn's disease. Various medical and surgical modalities have a meager complex fistula closure rate of 22–36% [6]. This may be due to the inherent aggressive nature of perineal Crohn's disease and possibly inadequate drainage. Initial responders to seton drainage and anti-TNF-alpha therapy experience up to 42% loss of response and up to 16% rate of fistula recurrence [7]. Despite their chronic and recurrent nature, the development of malignancy from Crohn's fistulae has been reported sporadically in the literature, consisting of case series and case reports [3, 4, 8, 9].

We report a rare case of SCC arising from an enterocutaneous fistula in a young female patient with 24 years of Crohn's disease, 10 of which was complicated by recurrent perineal fistulae. A systematic review of malignant transformation in perianal fistulae of Crohn's disease by Thomas et al. found an average 19-year duration of Crohn's disease and 9.5-year duration of fistula prior to detection of cancer [3]. Furthermore, there was a failure to detect cancer in 59% of the patients at the time of initial exam, with only 20% of the patients having physical characteristics suspicious for malignancy [3]. Our patient certainly falls within the observed statistics and validates the difficulty of diagnosing malignancy in this setting.

The causes of SCC in fistulizing perineal Crohn's have only been postulated. The pathophysiology of malignant degeneration of chronic wounds and irritation, however, has been elucidated in other specialties [10]. Chronic irritation and injury of a mucosal surface cause constant epithelial regeneration and ulceration, leading to dysplasia and eventual carcinoma. This has been shown in the setting of alcohol and smoking with esophageal squamous cell carcinoma and in chronic burn wounds with Marjolin's ulcers. It is possible that this model can explain the malignant conversion in patients with chronic Crohn's fistulae. A recent systematic review of anal SCC in inflammatory bowel disease postulates that the human papilloma virus (HPV) initiates carcinogenesis by infecting keratinocytes at inflamed areas and integrating oncogenes E6 and E7 into the host genome [11]. PCR studies of immunosuppressed anal SCC patients as a result of IBD treatment tested positive for high-risk HPV variants [11]. It is believed that the epithelialized surfaces of perianal fistulae provide an entry point for the HPV virus [11].

Other contributing mediators include antimetabolites, such as azathioprine, and anti-TNF-alpha drugs that are key to Crohn's maintenance and perineal fistula management [5]. Similarly increased rates of skin cancer can be seen in transplant patients undergoing long-term immunosuppressive therapy. Cardiac and renal transplant patients taking azathioprine were found to be more than twice as likely to develop skin SCC [10]. In Crohn's patients, more extensive use of these medications can delay wound healing and increase the risk of malignant transformation.

Crohn's disease is commonly believed to be a contraindication to ileal pouch-anal anastomosis due to increased rate of disease recurrence within the pouch. Abscesses, pouch strictures, and fistulas ultimately necessitate pouch excision. However, young and motivated Crohn's patients would prefer an ileal pouch-anal anastomosis over a permanent stoma and are willing to accept disease recurrence, reoperation, and pouch failure. Our patient may have been served best with an end ileostomy after her diagnosis of Crohn's disease with perianal fistulae.

Due to the rarity of this disease, there is no standard protocol for treatment. Shwaartz et al. reported their experience of treating nine patients with squamous cell carcinoma of perianal Crohn's fistula [12]. Two-thirds of the patients received neoadjuvant chemoradiation and did not require abdominoperineal resection (APR); however, one-third of the patients had recurrent/persistent disease ultimately requiring APR [12]. The success rate of Nigro protocol was 75% in their series, similar to anal SCC without Crohn's disease [12]. The authors concluded that there might be a benefit to performing resection in these patients in order to avoid the harmful effect of radiation, which could be added later if necessary [12]. Our patient was managed in a similar manner.

Based on the findings in this case report and others presented in the literature, a high degree of suspicion of malignancy should be present in patients with chronic painful, poor-healing fistulae, especially in the absence of infection. Pain and stricture commonly limit the utility of examinations of fistula tracts, and exam under anesthesia should not be delayed to allow curettage and biopsy to confirm the diagnosis.

## References

[1] Nielsen O. H., Rogler G., Hahnloser D., Thomsen O. Ø. (2009). Diagnosis and management of fistulizing Crohn's disease. *Nature Clinical Practice Gastroenterology and Hepatology*.

[2] Ky A., Sohn N., Weinstein M. A., Korelitz B. I. (1998). Carcinoma arising in anorectal fistulas of Crohn's disease. *Diseases of the Colon and Rectum*.

[3] Thomas M., Bienkowski R., Vandermeer T. J., Trostle D., Cagir B. (2009). Malignant transformation in perianal fistulas of crohn's disease: a systematic review of literature. *Journal of Gastrointestinal Surgery*.

[4] Lightdale C. J., Sternberg S. S., Posner G., Sherlock P. (1975). Carcinoma complicating Crohn's disease. Report of seven cases and review of the literature. *The American Journal of Medicine*.

[5] Pinto Pais T., Fernandes S., Carvalho J. (2014). Squamous cell carcinoma in enterocutaneous fistula associated with Crohn's disease: first case report. *Journal of Crohn's and Colitis*.

[6] Bor R., Farkas K., Bálint A. (2015). Efficacy of combined anti-TNF-alpha and surgical therapy in perianal and enterocutaneous fistulizing Crohn's disease—clinical observations from a tertiary Eastern European center. *Scandinavian Journal of Gastroenterology*.

[7] Sands B. E., Blank M. A., Patel K., van Deventer S. J. (2004). Long-term treatment of rectovaginal fistulas in Crohn's disease: response to infliximab in the ACCENT II study. *Clinical Gastroenterology and Hepatology*.

[8] Benjelloun E. B., Abkari M., Ousadden A., Ait Taleb K. (2013). Squamous cell carcinoma associated anal fistulas in Crohn's disease unique case report with literature review. *Journal of Crohn's and Colitis*.

[9] Buchman A. L., Ament M. E., Doty J. (1991). Development of squamous cell carcinoma in chronic perineal sinus and wounds in Crohn's disease. *The American Journal of Gastroenterology*.

[10] Coghill A. E., Johnson L. G., Berg D., Resler A. J., Leca N., Madeleine M. M. (2016). Immunosuppressive medications and squamous cell skin carcinoma: nested case-control study within the skin cancer after organ transplant (SCOT) cohort. *American Journal of Transplantation*.

[11] Slesser A. A. P., Bhangu A., Bower M., Goldin R., Tekkis P. P. (2013). A systematic review of anal squamous cell carcinoma in inflammatory bowel disease. *Surgical Oncology*.

[12] Shwaartz C., Munger J. A., Deliz J. R. (2016). Fistula-associated anorectal cancer in the setting of Crohn’s disease. *Diseases of the Colon & Rectum*.

